# Right-Sided Type IV Branchial Cleft Anomaly in a Seven-Year-Old Boy: A Case Report

**DOI:** 10.7759/cureus.86693

**Published:** 2025-06-24

**Authors:** Janvi J Shukla, Sara Morgan, David Wassef, Brian Manzi

**Affiliations:** 1 Otolaryngology - Head and Neck Surgery, Rutgers University New Jersey Medical School, Newark, USA

**Keywords:** branchial cleft anomaly, branchial cleft cyst, congenital neck mass, fourth branchial cleft cyst, head and neck mass

## Abstract

Type IV branchial cleft anomalies, which can present as a cyst, sinus, or fistula, are the rarest type of branchial cleft anomalies. Although they can present at any age, they typically present during childhood with a history of frequent neck abscesses. Type IV branchial cleft anomalies usually occur on the left side near the medial lower border of the sternocleidomastoid (SCM) muscle and can extend to the pyriform sinus in the larynx. They can also present with thyroid involvement. Imaging with ultrasound, computed tomography (CT), magnetic resonance imaging (MRI), sinogram, and direct laryngoscopy (DL) can aid in diagnosis. Sinus tracts, while not always present, may develop as a complication. Treatment is with surgical excision of the tract and debridement of the abscess in the setting of acute infection. Here, we present a rare case of right-sided type IV branchial cleft cyst in a seven-year-old boy who was treated primarily with ablation using Bugbee cauterization and drainage, avoiding the need for any further surgical intervention, including further drainage or thyroidectomy.

## Introduction

The branchial (pharyngeal) apparatus is a transient embryologic structure that develops during the fourth to seventh weeks of gestation and plays a key role in the formation of head and neck anatomy. It comprises six paired branchial arches, each separated externally by clefts (ectodermal invaginations) and internally by pouches (endodermal outpouchings). These structures contribute to the formation of various tissues, including cartilage, muscle, nerves, and vascular elements of the face and neck. [1]. Failure of proper obliteration or involution of these components can result in congenital anomalies such as cysts, sinuses, or fistulas, collectively referred to as branchial cleft anomalies [2-4].

Branchial cleft anomalies are one of the most frequently encountered neck masses in the pediatric population, accounting for about 20-30% of such lesions, with their location determined by the branchial cleft of origin [2-5]. There are four types (types I-IV) of branchial cleft anomalies, with type IV being due to failure of the fourth branchial cleft to involute and is considered the rarest, accounting for about 1-2% of all cases [3,6]. Type IV branchial cleft anomalies usually occur on the left side near the medial lower border of the sternocleidomastoid (SCM) muscle and may include a tract that can extend up to the pyriform sinus in the larynx [3].

They are present at birth but often go unnoticed until later in life, generally manifesting in childhood but can appear at any age. Usually, they present as a neck mass when they become inflamed secondary to an acute infection. Type IV branchial cysts appear as a firm mass or infected cyst draining to the skin of the neck or to the pyriform sinus [3]. Diagnosis is based on clinical and radiologic findings. Although there is no consensus on the preferred imaging study, ultrasound, computed tomography (CT), magnetic resonance imaging (MRI), and sinogram can all aid with better visualization of the cyst and its tract [2-3,5]. These imaging modalities would demonstrate a contrast-enhancing complex cystic lesion, possibly with septations anterior to or within the thyroid gland, with a tract into the larynx. The location and trajectory of these branchial cleft cysts aid in diagnosis; however, they are not confirmatory [7]. Diagnostic direct laryngoscopy (DL) has the best positive predictive value of 90% due to directly visualizing and identifying drainage pathways into the larynx [8].

Type IV branchial cleft anomalies can be confused with other rare entities such as hypopharyngeal pterygoid fistulas; however, the distinction lies in their embryologic origin and tract trajectory. Type IV branchial cleft anomalies typically involve a sinus tract originating from the apex of the pyriform sinus and extending through or near the thyroid gland. By contrast, hypopharyngeal pterygoid fistulas are not branchial in origin and follow a distinct path involving deeper hypopharyngeal structures and the pterygoid musculature [8-9].

Type IV branchial cleft anomalies are often linked with thyroid abnormalities such as acute suppurative thyroiditis and thyroid abscess because the branchial cleft tract can pass through the thyroid gland. Some have suggested that thyroid abscesses almost always arise from branchial cleft anomalies, especially with type IV anomalies [10]. This association can sometimes mask the underlying branchial cleft cyst, leading to a delay in diagnosis [11]. 

Treatment is with elective surgical excision. Risk of recurrence is about 3% but can be as high as 20% in the case of previous surgery or recurrent infections [3]. Conventional surgical techniques for type IV branchial cleft cysts involve an open standard transverse cervical incision with occasional thyroid lobectomy. However, surgery has a higher probability of complications or injury to cervical neurovascular structures, mainly in neonates and children aged 8 or younger [8]. Current trends are showing promising results with other less invasive options, such as endoscopic cauterization, a minimally invasive technique with fewer complications and lower cost [11-12]. In addition, a meta-analysis of 2,263 articles showed a comparable success rate after primary treatment with cauterization to surgery and concluded with endoscopic cauterization as the treatment of choice for third and fourth branchial pouch sinuses because of the lower morbidity rate [12].

Here, we present a rare case of right-sided type IV branchial cleft cyst in a seven-year-old boy treated successfully with endoscopic Bugbee cauterization, which required no further intervention, including repeat drainage or hemithyroidectomy.

## Case presentation

A seven-year-old boy with a past medical history of intermittent asthma and snoring who first presented to the emergency department (ED) with progressive fevers and sore throat over three weeks. He was diagnosed with an upper respiratory infection and discharged on ibuprofen. Two days later, the patient presented to the ED again for continued fevers and throat pain. Physical exam was significant for mild pharyngeal erythema and cervical lymphadenopathy. During both visits to the ED, he was negative for strep throat, flu, and COVID-19. He was discharged from the ED on a 10-day course of amoxicillin. After completing the antibiotic course, the fevers and throat pain persisted, and he returned to the ED, at which time he was positive for rhinovirus. He was treated with ibuprofen again and discharged. The next day, the patient presented to a different emergency department for the same symptoms and was started on azithromycin. 

**Figure 1:**
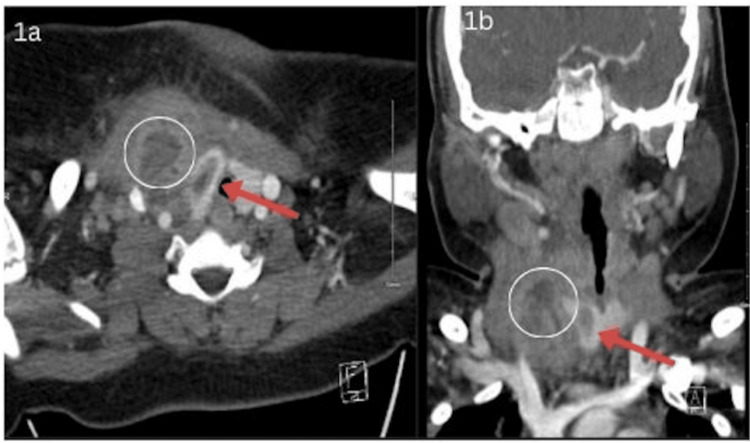
CT scan with contrast showing a 4 cm deep right neck abscess (circle) with an intrathyroidal component (red arrow) consistent with a type IV branchial cleft anomaly.

Two days later, he presented back to the ED, this time due to swelling on the anterior neck. Vital signs were significant for fever, tachycardia, and tachypnea. On physical exam, he had a 3 cm x 1 cm indurated erythematous mass on the anterior neck above the right suprasternal clavicular area and lymphadenopathy. There was no fluctuance of the mass, and he reported no neck stiffness or dysphagia. Labs are significant (Table 1) for elevated C-reactive protein (CRP) (9.9) and erythrocyte sedimentation rate (ESR) (93) and significant leukocytosis (22.5) with a neutrophilic predominance (74%). An ultrasound of the neck showed an irregular 4.2 x 3.4 x 3.1 cm structure in the right neck that extended to the thyroid gland and CT neck with contrast (Figure 1) showed a 4 cm right deep neck abscess with a right intrathyroidal component, consistent with the trajectory of a type IV branchial cleft cyst. He was subsequently started on IV vancomycin and transferred to our institution for further management. Based on clinical presentation and imaging, he was diagnosed with a type IV branchial cleft cyst with a deep abscess involving the thyroid. 

**Table 1:** Significant lab values CBC: complete blood count, HGB: hemoglobin, HCT: hematocrit, WBC: white blood cell, CMP: comprehensive metabolic panel, BUN: blood urea nitrogen, CRP: C-reactive protein, ESR: erythrocyte sedimentation rate

Lab	Patient value	Normal value
CBC		
HGB	8.9	12.0-14.8 g/dL
HCT	30.5	37.0-45.0%
WBC	22.5	3.6-11.8 10*3/uL
Neutrophils	74.0	32.0-54.0%
Granulocytes	1.0	<0.7%
Lymphocytes	14.0	35.0-65.0%
CMP		
Glucose	103	60-100 mg/dL
Calcium	9.6	8.7-10.4 mg/dL
Creatinine	0.5	0.7-1.3 mg/dL
BUN	12	9-23 mg/dL
Total bilirubin	0.2	0.3-1.2 mg/dL
Alkaline phosphatase	188	46-116 U/L
Albumin	3.9	3.2-4.8 g/dL
Inflammatory markers		
CRP	9.9	0.4-1.0 mg/dL
ESR	93	3-13 mm/hr

**Figure 2:**
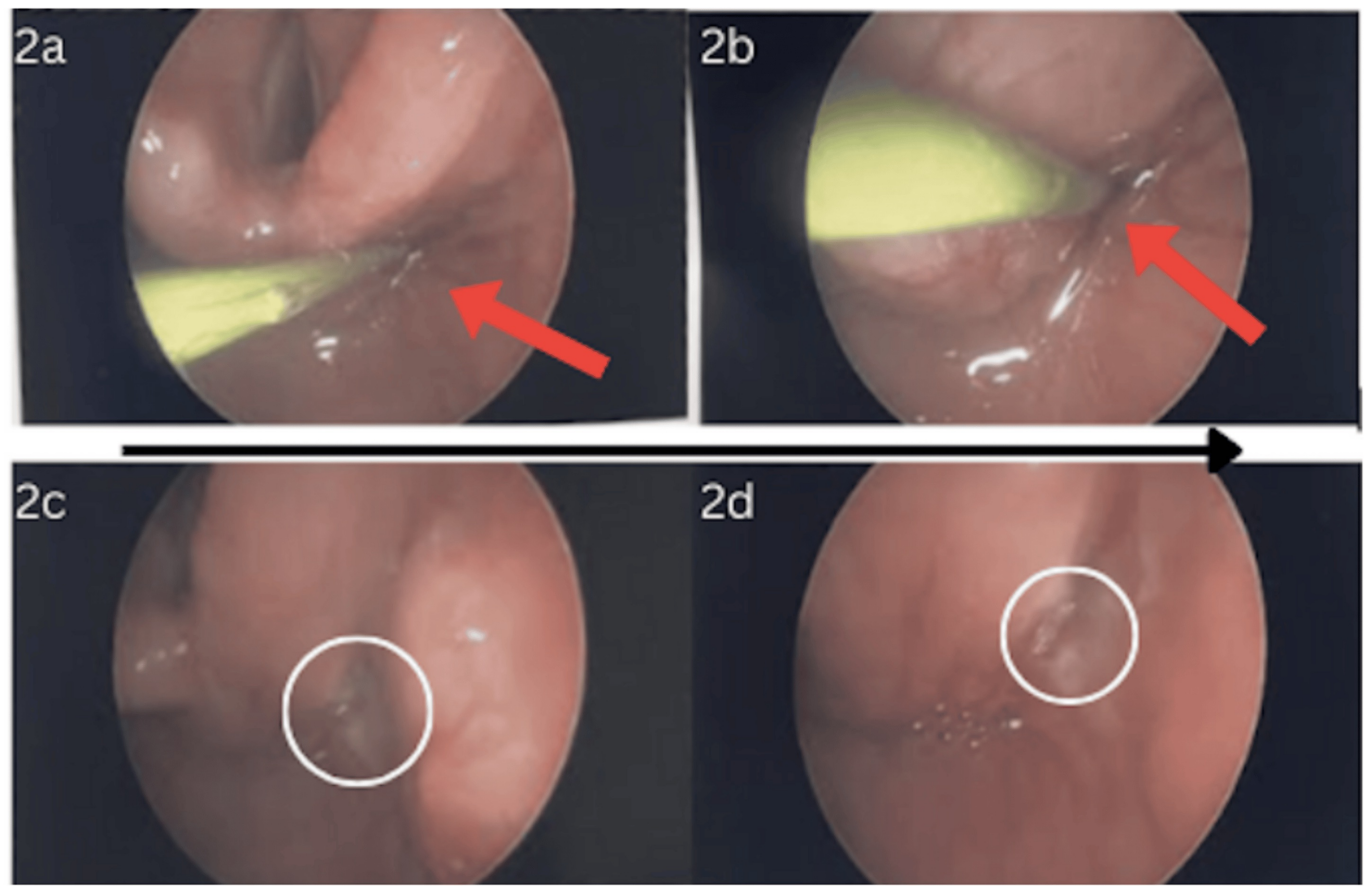
a-b) Direct laryngoscopy was used to visualize and ablate the branchial cleft tract opening at the apex of the right pyriform sinus. c-d) Post-cauterization shows successful ablation of the tract opening.

He was taken to the operating room the following day and underwent a direct laryngoscopy using a 0-degree endoscope, which revealed a tract in the apex of the right pyriform sinus, confirming the diagnosis of a type IV branchial cleft cyst. The tract was ablated using a monopolar flexible Bugbee and a laryngeal suction Bovie (Figure 2) to minimize complication risk. He also underwent incision and drainage of the right neck abscess. Contrary to commonly practiced techniques, we did not perform a thyroidectomy due to the acute infection affecting surgical planes in order to minimize the risk of injury to the recurrent laryngeal nerve, parathyroid glands, and surrounding great vessels. A ¼ inch Penrose drain was left in place for several days to facilitate drainage. At his two-week follow-up appointment, the patient was doing well with no complaints, recurrences of the neck mass, or complications. The patient was continuously followed at three months and nine months with normal physical examination with no return of infection or mass. In addition, he had a normal thyroid ultrasound and thyroid-stimulating hormone levels at these visits, thus avoiding further aggressive surgical intervention, including thyroid lobectomy. 

## Discussion

Type IV branchial cleft anomalies are the rarest version of branchial cleft anomalies, with approximately 40 cases reported in the literature since 1972 [8,13]. Owing to its rarity, type IV branchial cleft anomalies are often hard to detect and are misdiagnosed. As a result, it is important to consider this anomaly as a differential diagnosis of lateral neck masses [7]. However, on the initial presentation, a neck mass may not be apparent, and the patient may present with non-specific symptoms, as in our case. 

Type IV branchial cleft anomalies are hypothesized to result from the failure of obliteration of the pharyngeal branchial duct and the fourth branchial cleft during development [3,6,7]. Type IV anomalies occur near the medial lower border of the SCM, most commonly on the left side. Only about 6% of type IV anomalies have been reported to be right-sided [3,14]. Our patient had this rare laterality, emphasizing the importance of being aware of this uncommon presentation. In the pediatric population, they generally occur as sinuses or fistulas, while in adults, cysts are more common [11,15]. If a tract is present, it generally passes deep to the common carotid and loops around either the aortic arch or subclavian, runs superficially to the recurrent laryngeal nerve and hypoglossal nerve, and terminates in the apex of the pyriform sinus in the larynx [3]. 

Type IV branchial cleft anomalies classically present as a neck mass when there is inflammation due to an infection resulting from retrograde transmission of hypopharyngeal organisms [3,10]. In most cases, patients report a repetitive history of such infections, causing recurrent abscesses in the neck [15]. Contrary to typical presentations, our patient initially only presented with fevers and a sore throat, with no documentation of a neck mass until further progression of the infection. 

Type IV anomalies are also linked with thyroid abnormalities such as acute suppurative thyroiditis and thyroid abscess. Due to this association, at times when a thyroid abscess is suspected, it can mask the underlying branchial cleft cyst, causing an average delay of six years in diagnosis [11]. However, it is important to consider that thyroid abscesses most commonly arise in the setting of branchial cleft anomalies [10]. This occurs because the sinus tract typically passes through the thyroid tissue, causing the abscess from the skin or pyriform sinus to involve the ipsilateral thyroid lobe [7,15]. 

For diagnosis, imaging with ultrasound, CT, MRI, and sinogram can all be useful. Although there is no consensus on the preferred imaging study, a DL and barium esophagram have been the most useful diagnostic method [2,3,5,7]. A DL can be particularly helpful in visualizing the opening of the sinus tract into the apex of the pyriform sinus in the setting of inflammation [7,15]. In our patient, we had done a DL intraoperatively, which showed the tract at the apex of the piriform sinus. Imaging was also done preoperatively, which showed an abscess with an intrathyroidal component and sinus tract, further aiding in diagnosis. 

In considering treatment, complete surgical excision of the entire cyst and tract is the gold standard. If there is an abscess present, an incision and drainage should be performed to control the infection [16]. It is important to excise the entire tract, preferably during a quiescent period, to avoid recurrence [13]. However, excision can also occur during acute infection along with the incision and drainage of the abscess. 

In the case of thyroid involvement, there is a consensus that the involved portion of the thyroid adjacent to the fistula should be removed due to the high risk of severe infection or primary carcinoma [7,8,10,15]. Our patient did not require a thyroidectomy and had a normal thyroid US on post-op follow-up in the clinic. 

Various methods for surgical treatment have been described, including an open approach with a transverse cervical incision, endoscopy-assisted trans-oral resection, and CO2 laser excision with satisfactory results [7,8,15,17]. Currently, endoscopy with cauterization, which is the technique we used for our patient, is one of the most employed techniques. It has the advantage of offering a minimally invasive technique with minimal complications and costs, higher scar satisfaction, and similar surgical success compared to the open approach [8,7,11,15]. It has also been suggested that in children, up to the age of 8, it is preferable to emphasize medical treatment and delay surgical neck exploration due to a higher risk of complications in that patient population [8]. In our case, the use of Bugbee cauterization offered the additional advantage of avoiding the need of further intervention, including repeat drainage or hemithyroidectomy. 

Recurrence risk following treatment of type IV branchial cleft cysts varies depending on the technique used, the completeness of tract removal, and associated infections. While surgical excision followed by partial thyroidectomy has the lowest recurrence rate of 8% and is considered curative in most cases, children aged eight or younger showed the highest risk of complications. Endoscopic cauterization, including Bugbee ablation, has demonstrated favorable outcomes with low recurrence rates (15%) and minimal complications [8]. Sun et al. reported no recurrence in 21 of 23 pediatric patients treated with endoscopic electrocauterization, with a mean follow-up of 7.4 years [18]. Moreover, a study done by Verret et al. observed no recurrences in seven out of 10 children with branchial cleft cysts over an average of three years of follow-up post-endoscopic cauterization [19]. Similarly, Watson et al. reported no recurrence in all five pediatric patients managed endoscopically over a mean follow-up period of 25 months [20]. Based on these findings, it is recommended that patients undergo follow-up at two weeks postoperatively to assess for wound healing and early complications, followed by evaluations at three months, six months, and 12 months. Continued annual follow-up for up to two to five years may be warranted in high-risk cases, particularly when the sinus tract traverses the thyroid or when intraoperative visualization of the tract is limited. Our patient was monitored at two weeks, three months, and nine months postoperatively, with no recurrence or thyroid abnormalities, supporting the efficacy of endoscopic Bugbee cauterization and a structured follow-up protocol.

## Conclusions

Type IV branchial cleft cysts are a rare entity that often go undiagnosed. Although more commonly seen on the left side, it is important for clinicians to recognize that they can also occur on the right side. Incision and drainage of the abscess and endoscopic Bugbee cauterization of the tract can be an effective treatment option, which avoids the need for a thyroidectomy and achieves remission. Future research should aim to establish a structured treatment plan that incorporates minimally invasive techniques when suitable and a follow-up protocol for pediatric patients presenting with type IV branchial cleft cysts.
